# Ecological differentiation of members of the *Culex pipiens* complex, potential vectors of West Nile virus and Rift Valley fever virus in Algeria

**DOI:** 10.1186/s13071-016-1725-9

**Published:** 2016-08-17

**Authors:** Raouf Amara Korba, Moufida Saoucen Alayat, Lazhari Bouiba, Abdelkarim Boudrissa, Zihad Bouslama, Slimane Boukraa, Frederic Francis, Anna-Bella Failloux, Saïd Chaouki Boubidi

**Affiliations:** 1 Laboratoire Ecologie des Systèmes Terrestres et Aquatiques, Département de Biologie, Faculté des Sciences, Université Badji Mokhtar, Annaba, Algérie; 2 Institut Pasteur d’Alger, Unité d’Entomologie Médicale, Service d’Eco-épidémiologie parasitaire et génétique des populations, Alger, Algérie; 3 Laboratoire de Biologie Animale Appliquée, Faculté des Sciences, Département de Biologie, Université Badji Mokhtar, Annaba, Algérie; 4 Unit of Functional and Evolutionary Entomology, Gembloux Agro-Bio Tech, University of Liège, Passage des Déportés 2, 5030 Gembloux, Belgium; 5 Institut Pasteur, Department of Virology, Arboviruses and Insect Vectors, Paris, France

**Keywords:** *Culex pipiens* complex, Molestus, CQ11, Hybrid, Algeria, Autogeny, Stenogamy

## Abstract

**Background:**

We investigated the ecological differentiation of two members of the *Culex pipiens* complex, *Cx. p. pipiens* form pipiens and *Cx. p. pipiens* form molestus in three sites, El-Kala, M'Sila and Tinerkouk in Algeria. These two forms are the most widespread mosquito vectors in temperate regions exhibiting important behavioural and physiological differences. Nevertheless, this group of potential vectors has been poorly studied, particularly in North Africa.

**Methods:**

Ten larval populations of *Cx. p. pipiens* were sampled from various above- and underground habitats in three zones representing the three bioclimatic regions in Algeria. The reproduction characteristics were also investigated in the laboratory to define the rates of autogeny and stenogamy. Identification of *Cx. p. pipiens* members present in Algeria was achieved using a molecular analysis with the microsatellite CQ11 locus.

**Results:**

We detected larvae of *Cx. p. pipiens* in all areas suggesting that the species is a ubiquitous mosquito well adapted to various environments. To our knowledge, this study provides the first molecular evidence of the presence of the *Cx. p. pipiens* form molestus and hybrids (molestus/pipiens) in Algeria with a high proportion of molestus form (48.3 %) in comparison with hybrids (36.8 %) and pipiens form (14.9 %).

**Conclusions:**

Some unexpected correlations between the proportion of forms pipiens, molestus and hybrids, and mosquito biological characteristics were observed suggesting some epigenetic effects controlling *Cx. p. pipiens* mating and reproduction. Consequences for pathogen transmission are discussed.

**Electronic supplementary material:**

The online version of this article (doi:10.1186/s13071-016-1725-9) contains supplementary material, which is available to authorized users.

## Background

*Culex pipiens pipiens* is one of the most important mosquito species in terms of geographical distribution and ability to transmit pathogens [1]. In North Africa, *Cx. p. pipiens* is a competent vector of several pathogens infecting animals and humans including West Nile virus (WNV) and Rift Valley fever virus (RVFV) [2–5]. In Algeria, *Cx. p. pipiens* is the most widespread mosquito species [6–9], and an efficient vector of WNV and to a lesser extent, of RVFV in experimental conditions [5].

The first isolation of WNV in Algeria was in Djanet, southeast of the country in 1968 [10]. The virus was isolated from a pool of 215 mosquitoes belonging to the genus *Culex*. In 1994, the virus appeared again causing an outbreak with a total of 50 human cases including eight deaths in Tinerkouk (Adrar Department, southeast Algeria) [11]. From neighbouring countries, other human and equine cases were reported in Tunisia in 1997, while only equine cases were detected in 1996 in Morocco [12, 13]. In the following years, other human cases were reported: in Morocco in 2003 [14] and in Tunisia between 2003 and 2012 [15, 16] suggesting that WNV is still circulating within the region. In western Africa, a major RVF outbreak occurred, close to Algeria, in Mauritania and in Senegal in 1987 and resulted in 220 human deaths [17]. Moreover, a recent study identified the Maghreb region as high-risk countries for RVF emergence [18].

*Culex p. pipiens* exists in two forms or biotypes that exhibit substantial differences in both behavioural and physiologic characteristics but are morphologically indistinguishable [19]. In Europe, *Cx. p. pipiens* form molestus is considered as stenogamous (mates in confined spaces), autogenous (able to lay its first batch of eggs without a blood meal), prefers underground water bodies with high organic contents and does not diapause. In contrast, *Cx. p. pipiens* form pipiens is considered as eurygamous (mates while swarming in open areas), anautogenous (requires a blood meal to lay eggs), overwinters (enters reproductive diapause) and colonizes a wide variety of aboveground breeding sites [20]. Several studies suggest that both forms have different trophic preferences: form pipiens biting mainly birds and form molestus feeding on mammals including humans, whereas hybrids exhibit an opportunistic behaviour and can readily feed on both hosts [21]. However, several studies also mention a potential local variation in host selection depending on host availability [22]; these feeding behaviours are thought to influence the transmission of avian and mammalian pathogens [21].

For a better implementation of vector control measures, molecular identification, biological and ecological characterisation of the *Cx. p. pipiens* forms present in Algeria is of prime importance. In this study, we used the microsatellite locus CQ11 to analyse samples of *Cx. p. pipiens* collected in both aboveground and underground habitats in three bioclimatic regions of the country (humid, sub-arid and arid). Reproductive strategies of *Cx. pipiens* complex forms were compared for a better understanding of their adaptability according to their environment.

## Methods

### Study area

**Fig. 1:**
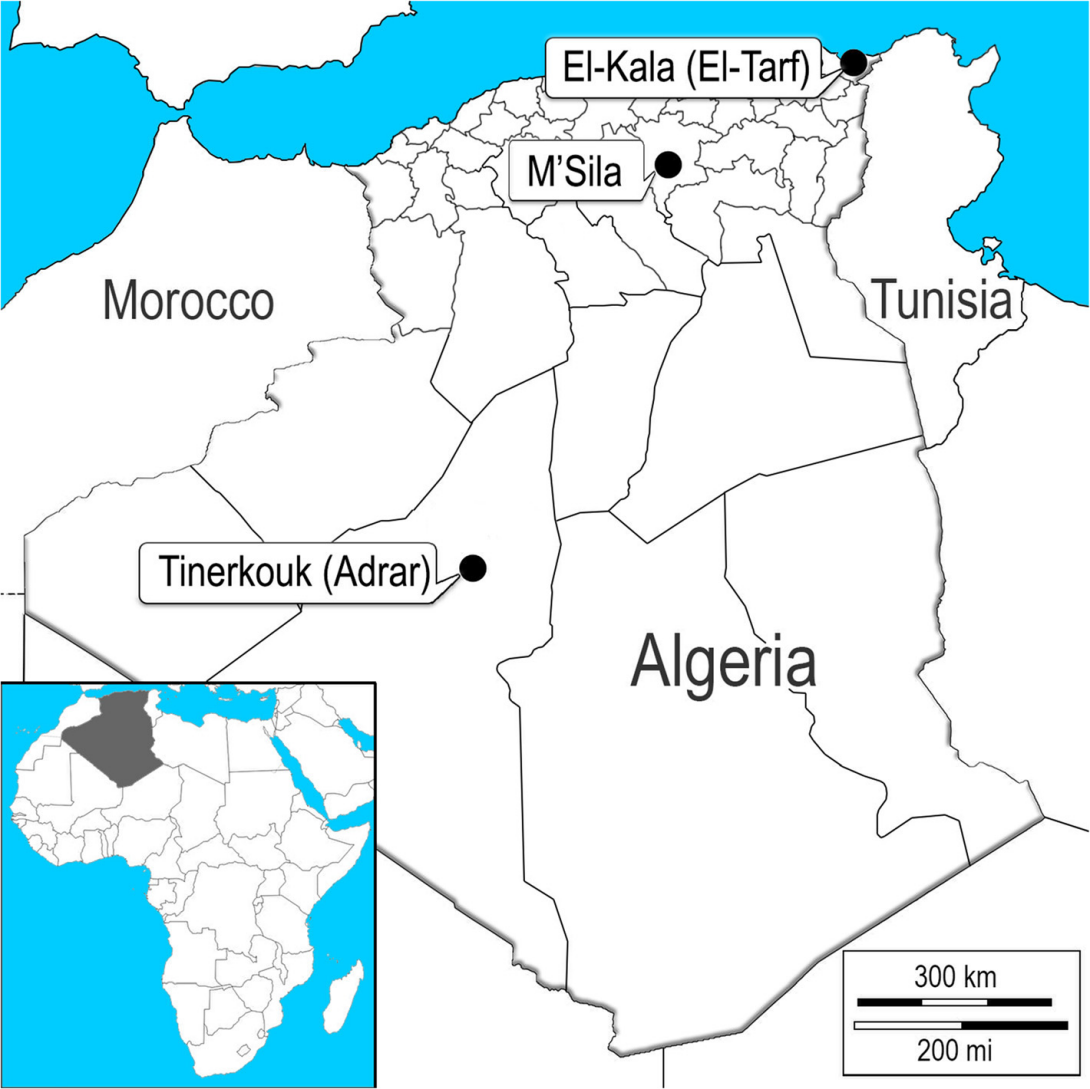
Localization of *Cx. p. pipiens* samples collected in 2010 in Algeria

**Fig. 2:**
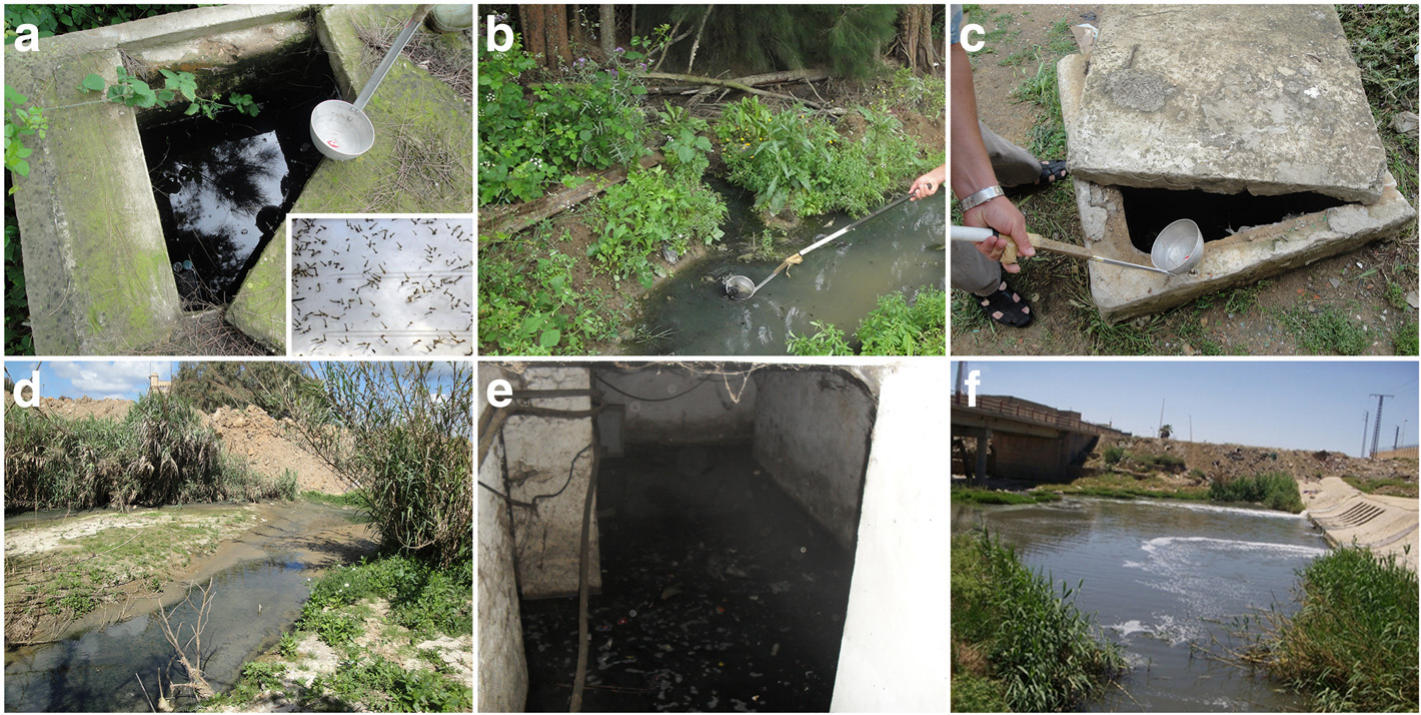
Different types of breeding sites where *Cx. p. pipiens* populations were sampled. **a** Underground-rural El-Kala. **b** Aboveground-rural El-Kala. **c** Underground sub-urban El-Kala. **d** Aboveground urban El-Kala. **e** Underground urban El-Kala. **f ** Aboveground sub-urban M’Sila

Algeria is divided into three major bioclimatic regions based on the classification of Emberger [23]: humid, sub-arid and arid. Three sites were selected: El-Kala in the humid region El-Tarf department; (36°53'N, 8°26'E) with pluviometry: 900–1200 mm/year, M’Sila department (5°7'N, 4°33'E) and pluviometry: 300–600 mm/year, and Tinerkouk in the arid desert region at Adrar department; (29°42'N, 0°43'E) with pluviometry: < 100 mm/year (Fig. 1). The area of El-Kala was selected partly because of the high diversity of potential breeding sites for *Cx. p. pipiens* and their proximity to relatively dense human populations and other hosts including migratory birds, suggesting that it could be a hotspot for WNV circulation. The department of M’Sila is characterized by an intermediate climate between the north and the south of the country where the inhabitants complain regularly about an important nuisance caused by mosquitoes. The area of Tinerkouk was selected owing to its arid climate and because this city has experienced a WNV outbreak in 1994. In each bioclimatic region, different habitats were chosen: urban (centre of the city), sub-urban (outskirts of the city) and rural (outside the city). In each habitat, aboveground and underground breeding sites were examined if available. Six categories of larval habitat were then selected (e.g. Fig. 2).

### Mosquito collections

A total of 10 sites were classified according to the habitat (urban, suburban or rural) and the type of breeding site (aboveground or underground) (Table 1). Fourth instar larvae were sampled from May to October 2010 using the dipping method [24] with ten dips in different places of the breeding site. Larval density was expressed as numbers of larvae per litre and estimated according to a range of five classes: class 1 for larval density = 0, class 2 for larval density = 10 (1–10), class 3 for larval density = 50 (11–50), class 4 for larval density = 100 (51–100), class 5 for larval density = 500 (> 100). Ecological data characterizing each breeding site were recorded: the presence of vegetation (Additional file 1: Table S1), temperature, pH, salinity and percentage of O_2_ (Table 1). The water of each breeding site was analysed for BOD5 (Biological Oxygen Demand) to estimate the concentration of organic matter and evaluate the correlation with larval densities.

**Table 1:** Chemical and physical properties of the larval habitats of *Cx. p. pipiens*

Site	Habitat	Breeding sites	Mean density of larvae	pH	Temperature (°C)	Salinity (g/l)	O^2^ (%)	BOD5 (mg/l)
El-Kala	Urban	Underground	66	7.3	21.1	0.5	1	190
		Aboveground	255	7.8	21.4	0.4	1	6
	Sub-urban	Underground	340	7.8	19.2	0.7	0	3
		Aboveground	38	8	19.1	0.6	0	28
	Rural	Underground	160	8.3	20.2	0.9	1	6
		Aboveground	380	7.9	26.4	0.8	0	32
M’Sila	Urban	Underground	27	7.9	19.7	1.1	1	7
	Sub-urban	Aboveground	165	8.1	20.3	1.8	0	12
	Rural	Aboveground	165	8.1	24.0	1.7	1	32
Tinerkouk	Urban	Underground	13	7.4	28.6	0.8	0.5	384

BOD5, Biological Oxygen Demand

### Reproduction characteristics

Larvae collected from each breeding site were sorted and reared in an insectarium at 26 ± 2 °C and relative humidity of 70 % ± 10 %; they were placed in plastic pans (18 × 25 × 5 cm) at a concentration of 200 larvae per litre and were fed with commercial cat food (Purina ONE®). Pupae were isolated individually in glass tubes with 10 ml of distilled water until emergence of adults. Fourty pairwise males and females were constituted for each breeding site and maintained for 3–5 days under a 12 h: 12 h LD photoperiod and a relative humidity of 60-80 % in 500 ml cardboard cups covered with a mesh screen and a piece of cotton soaked with 10 % sugar solution was provided as nutrient. Then, half of females (20) were removed using a mouth aspirator and placed in a cage (25 × 25 × 25 cm) covered with a mesh screen and males were removed and stored at -20 °C for molecular identification. Those females were deprived of sugar solution for 24 h before being exposed to a quail (*Coturnix japonica*) maintained in a small metal cage (20 × 10 × 10 cm). The quail was provided for one hour/day during 3 consecutive days until most of mosquitoes had successfully obtained a blood meal. Engorged females were individually maintained in cardboard cups and provided with sugar solution. The other half of females (20) were only supplied with a sugar solution and a cup of water was provided for egg laying. Several characteristics were then evaluated: autogeny/anautogeny, stenogamy/eurygamy, fecundity, fertility, and molecular identification.

#### Autogeny/anautogeny

Females able to lay eggs without any blood meal were qualified as autogenous (AU) and those which required a blood-meal as anautogenous (AN). The number of eggs laid by each female was counted. Number of specimens tested in each population (*n* = 20).

#### Stenogamy/eurygamy

Females able to mate in cups and capable of laying fertile eggs were considered as stenogamous. In contrast, females unable to be inseminated in confined spaces (laid sterile eggs) were qualified as being eurygamous. Number of specimens tested in each population (*n* = 40).

#### Fecundity and fertility

The fecundity rate corresponded to the number of eggs laid per female. Fertility rate was estimated by the proportion of larvae hatched from laid eggs. These proportions were compared between females which received a blood meal and those that received only sugar solution. Number of specimens tested in each population (*n* = 40).

### Molecular identification

*Culex p. pipiens* mosquito populations belonging to the 10 studied sites were classified according to their phenotypes (autogenous/anautogenous, stenogamous/eurygamous) and then we performed a correlation between obtained phenotypes and the genotypes of those populations assessed by multiplex PCR assay described in Bahnck & Fonseca (2006) [25]. A leg was placed directly into the reaction mixture containing primers amplifying the microsatellite CQ11 locus used to distinguish between the two forms of *Cx. pipiens* complex: pipiens and molestus (Additional file 2: Figure S2). The primers used were: pipCQ11R 5′-CAT GTT GAG CTT CGG TGA A-3′ and molCQ11R 5′-CCC TCC AGT AAG GTA TCA AC-3′. The forward primer CQ11F2 5′ GAT CCT AGC AAG CGA GAA C-3′. The DNA fragment size amplified varied between pipiens and molestus allowing us to distinguish the two forms in a single PCR reaction. Polymerase chain reaction (PCR) >products were run on a 2 % agarose gel. The pipiens and molestus forms presented PCR products of 180 bp and 250 bp, respectively. Hybrids exhibited both amplicons (180 bp/250 bp).

### Data analysis

Chi-square test was used for comparisons of rates (autogeny, stenogamy and the molecular forms of *Cx. p. pipiens*), Kruskal-Wallis H test to study the spatial variation of rates (autogeny, stenogamy) and Mann-Whitney test to evaluate the influence of blood meal on fecundity and fertility rates. All statistical tests were conducted using the IBM® SPSS® Statistics for windows v.21 (IBM Corp. Armonk, NY). *P*-values > 0.05 were considered non-significant.

## Results

All habitats investigated were colonized by *Cx. p. pipiens*.

### *Culex p. pipiens* is sensitive to high levels of organic matter

The chemical and physical properties of breeding sites were analysed as factors that could influence larval development (Additional file 3: Figure S1). pH, temperature, salinity, and dissolved oxygen exhibited comparable values without any relation with larval densities (Table 1). The density of larvae increased when the BOD5 decreased. The highest densities of larvae were found in the three breeding sites in the city of El-Kala corresponding to low levels of BOD5 (Table 1). The highest level of BOD5 was recorded in an urban underground breeding site in Tinerkouk, which was associated with a low larval density and the highest temperature of breeding site (Table 1).

### Autogeny/anautogeny

**Fig. 3:**
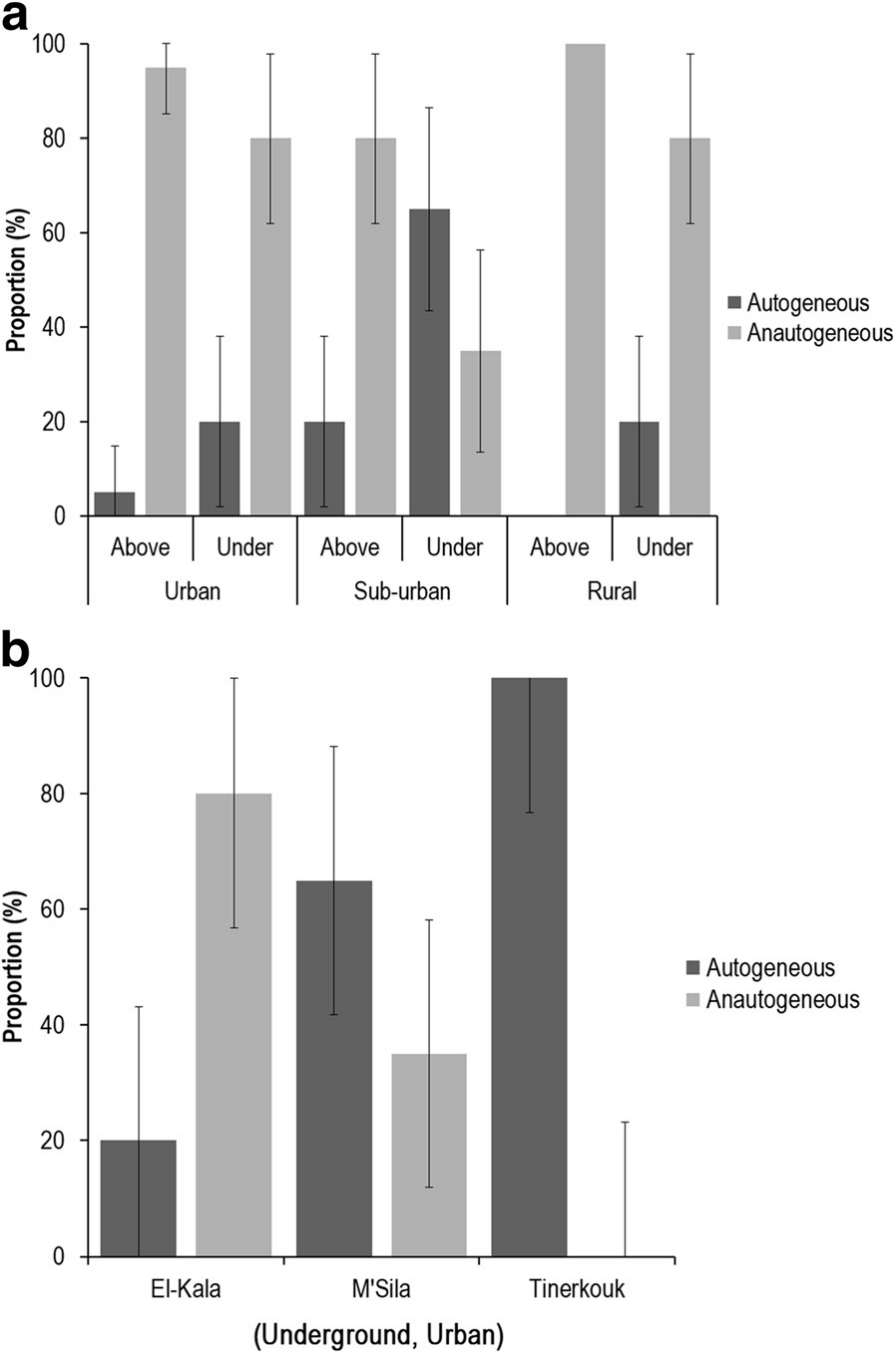
Proportions of autogenous/anautogenous *Cx. p. pipiens* collected in El-Kala (**a**) and in underground breeding sites in urban environments (**b**). Error bars indicate the 95 % confidence interval

To define whether autogenous mosquitoes were more prevalent in underground sites, we examined mosquitoes collected in El-Kala and found that autogenous mosquitoes were mostly a minority in breeding sites without any clear-cut relation with the habitat (above- or underground) (Fig. 3a). When considering a given habitat (urban, sub-urban or rural), the highest proportion of autogenous mosquitoes were found in underground breeding sites [Chi-square test: urban (*χ*^2^ = 2.054, *df* = 1, *P* = 0.151), sub-urban (*χ*^2^ = 8.286, *df* = 1, *P* = 0.004) and rural (*χ*^2^ = 4.444, *df* = 1, *P* = 0.035)]. When only analysing underground breeding sites in urban habitats of the three sites, El-Kala, M’Sila and Tinerkouk, the proportion of autogenous mosquitoes increased from El-Kala to Tinerkouk (Fig. 3b) (Kruskal-Wallis H test: *χ*^2^ = 26.761, *df* = 2, *P* < 0.0001).

### Stenogamy/Eurygamy

**Fig. 4:**
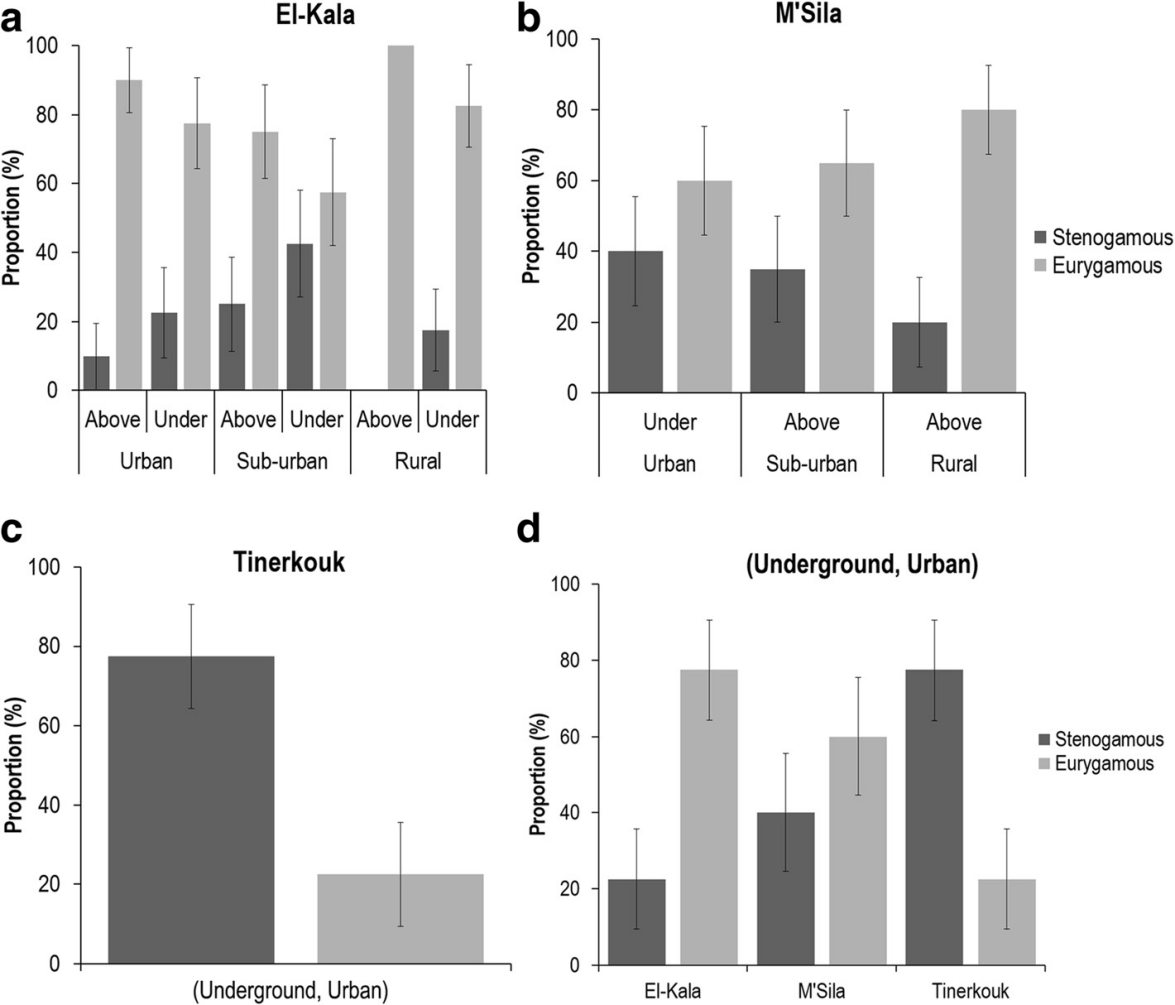
Proportions of stenogamous / eurygamous *Cx. p. pipiens* collected in El-Kala (**a**), M’Sila (**b**), Tinerkouk (**c**), and in underground breeding sites in urban environments (**d**). Error bars indicate the 95 % confidence interval

Eurygamous mosquitoes predominated in El-Kala (Fig. 4a); Chi-square test: urban (*χ*^2^ = 8.286, *df* = 1, *P* = 0.004), sub-urban (*χ*^2^ = 36.450, *df* = 1, *P* < 0.0001), rural (*χ*^2^ = 54.450, *df* =1, *P* < 0.0001) and in M’Sila (Fig. 4b) urban (*χ*^2^ = 1.600, *df* = 1, *P* = 0.206), rural (*χ*^2^ = 14.400, *df* = 1, *P* < 0.0001) and sub-urban (*χ*^2^ = 3.600, *df* = 1, *P* = 0.058) whatever the breeding site (above- or underground). Conversely, stenogamous mosquitoes predominated in the underground urban site of Tinerkouk (Fig. 4c) (Chi-square test: *χ*^2^ = 12.100, *df* = 1, *P* = 0.001). When considering the given habitat (urban, sub-urban or rural), the highest proportion of stenogamous mosquitoes were found in underground breeding sites in El-Kala, especially in rural habitats (Chi-square test: *χ*^2^ = 7.671, *df* = 1, *P* = 0.006), but not in M’Sila. When only considering underground urban sites, the proportion of stenogamous mosquitoes increased significantly from El-Kala to Tinerkouk (Fig. 4d) with a tendency similar to the proportion of autogenous females (Fig. 3b) (Kruskal-Wallis H test: *χ*^2^ = 25.168, *df* = 2, *P* < 0.0001).

### Fecundity and fertility

**Fig. 5:**
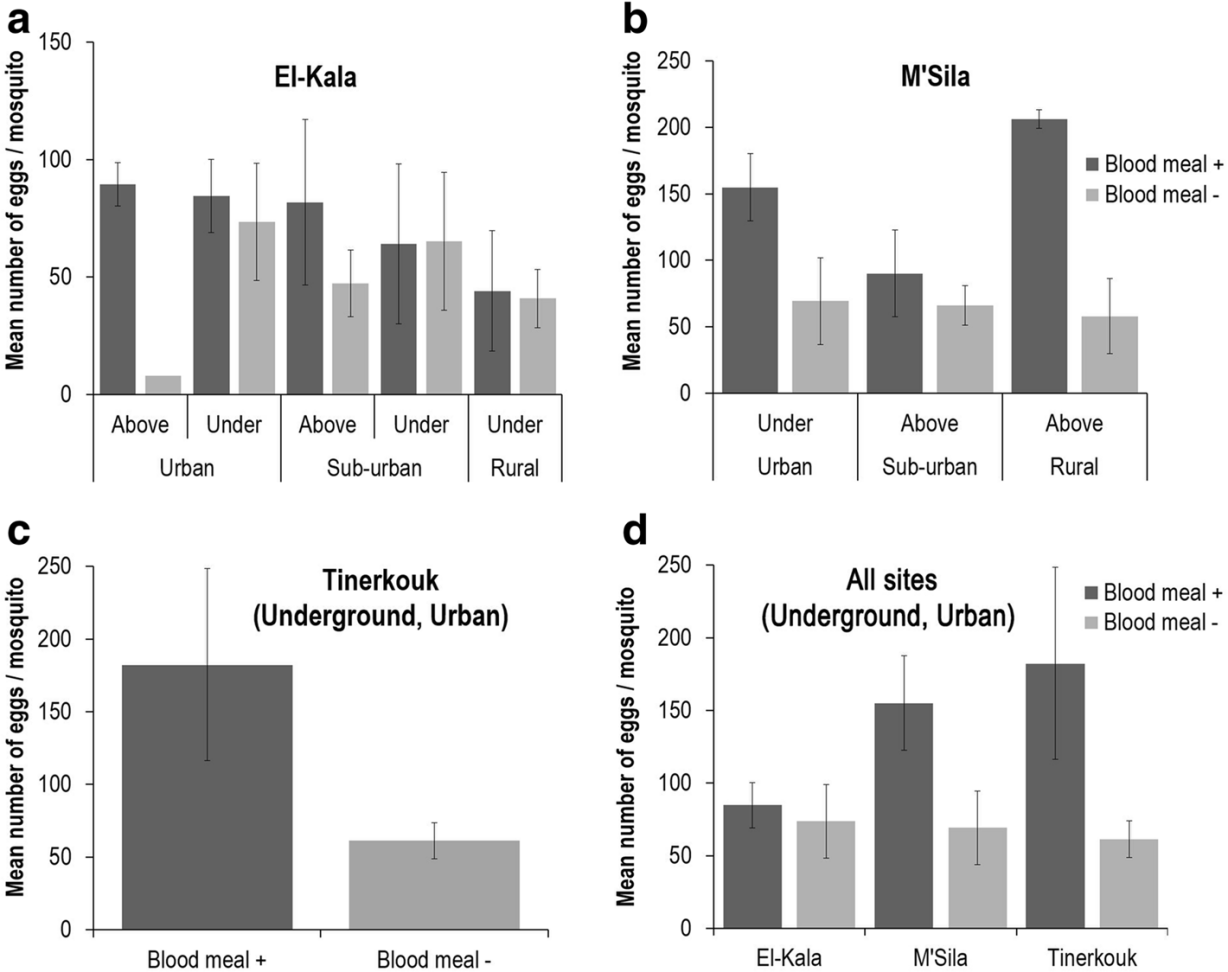
Fecundity expressed by the mean number of eggs laid by mosquitoes collected in El-Kala (**a**), M’Sila (**b**), Tinerkouk (**c**), and in underground breeding sites in urban environments (**d**). Error bars indicate the 95 % confidence interval

**Fig. 6:**
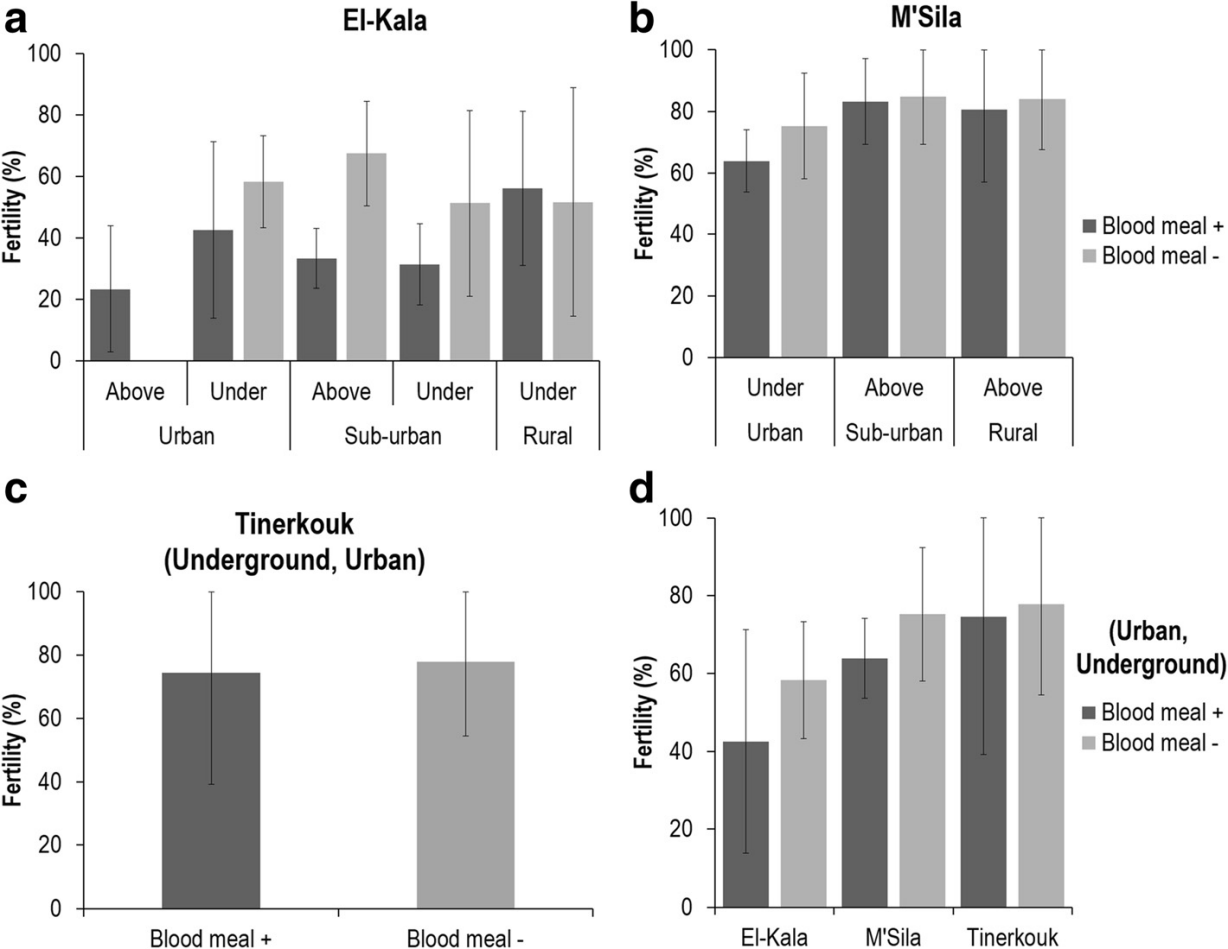
Fertility corresponding to the mean number of eggs hatched from eggs laid by mosquitoes collected in El-Kala (**a**), M’Sila (**b**), Tinerkouk (**c**), and in underground breeding sites in urban environments (**d**). Error bars indicate the 95 % confidence interval

To examine whether the mosquito status autogenous/anautogenous affects the number of eggs laid, we estimated the fecundity corresponding to the number of laid eggs per female and the fertility referring to the hatching success of laid eggs. Blood feeding increased the number of eggs laid by mosquitoes collected in El-Kala (Fig. 5a; Mann-Whitney test: *U* = 218, *P =* 0.028), M’Sila (Fig. 5b; Mann-Whitney test: *U* = 19.5, *P* < 0.0001) and Tinerkouk (Fig. 5c; Mann-Whitney test: *U* = 23, *P* < 0.0001). When only considering blood-fed mosquitoes from underground urban sites, the fecundity increased from El-Kala to Tinerkouk (Kruskal-Wallis H test: *χ*^2^ = 8.062, *df* = 2, *P* = 0.018), whereas it remained constant for unfed mosquitoes (Fig. 5d). When analysing fertility, blood feeding did not significantly affect the hatching success of eggs collected in El-Kala (Fig. 6a), M’Sila (Fig. 6b) and Tinerkouk (Fig. 6c). When only considering mosquitoes from underground urban sites, the fertility slightly increased from 10 % for El-Kala to 20 % for Tinerkouk whether mosquitoes were fed or unfed (Fig. 6d).

### Molecular identification

A total of 87 adults were characterized by PCR and frequencies of different forms are represented in Table 2. Overall, 14.9 % of the adults tested belonged to the pipiens form, 48.3 % to the molestus form and the remaining 36.8 % corresponded to hybrids (Additional file 2: Figure S2). Most breeding sites examined contained the three genetic forms of *Cx. p. pipiens* with a predominance of the molestus form. The pipiens form showed a preference for aboveground habitats (Chi-square test: *χ*^2^ = 6.100, *df* = 1, *P* = 0.013), while the molestus form and hybrids did not present any preference for aboveground or underground habitats [Chi-square test: molestus (*χ*^2^ = 0.857, *df* = 1, *P* = 0.355), hybrids (*χ*^2^ = 0.50, *df* = 1, *P* = 0.48)]. As expected, pipiens f. were mainly anautogenous/eurygamous, but molestus f. were at 78.6 % autogenous/stenogamous and hybrids presented several reproductive characteristics (Table 3). Thereby, only 6 (18.7 %) hybrids issued from Tinerkouk have expressed an autogeneous behaviour while 81.3 % were anautogenous from other sites (Table 3).

**Table 2:** Frequencies of different forms of the *Culex pipiens* complex in Algeria

Site	Habitat	Breeding sites	Frequency (%)		
			pipiens	molestus	hybrids
El-Kala	Urban	Underground	0	4 (44.4)	5 (55.5)
		Aboveground	4 (40)	3 (30)	3 (30)
	Sub-urban	Underground	0	7 (100)	0
		Aboveground	1 (12.5)	2 (25)	5 (62.5)
	Rural	Underground	1 (10)	8 (80)	1 (10)
		Aboveground	1 (14.3)	4 (57.1)	2 (28.6)
M’Sila	Urban	Underground	0	2 (25)	6 (75)
	Sub-urban	Aboveground	1(16.7)	5 (83,3)	0
	Rural	Aboveground	4 (33.3)	4 (33.3)	4 (33.3)
Tinerkouk	Urban	Underground	1(10)	3 (30)	6(60)
Total			13 (14.9)	42 (48.3)	32 (36.8)

**Table 3:** Frequencies (%) of different forms of the *Culex pipiens* complex in Algeria related to their phenotype status

Underground sites						Aboveground sites				
Form	AU/ST	AU/EU	AN/ST	AN/EU	TOTAL	AU/ST	AU/EU	AN/ST	AN/EU	TOTAL
pipiens	0	0	0	100 (*n* = 2)	2	0	0	0.091 (*n* = 1)	90.9 (*n* = 10)	11
molestus	62.5 (*n* = 15)	4.2 (*n* = 1)	33.3 (*n* = 8)	0	24	77.8 (*n* = 14)	16.7 (*n* = 3)	0	5.5 (*n* = 1)	18
hybrid	22.2 (*n* = 6)	0	16.7 (*n* = 3)	61.1 (*n* = 11)	18	0	0	35.7 (*n* = 5)	64.3 (*n* = 9)	14

## Discussion

This study corroborates that the human-biting *Culex pipiens pipiens* is a ubiquitous mosquito well adapted to a wide range of environments through the expression of a biological plasticity for mating and reproduction. Intriguingly, whatever the habitat (urban, sub-urban or rural) and the type of breeding site (above- and underground), the proportion of autogenous and stenogamous mosquitoes increased from El-Kala in the humid bioclimatic region to Tinerkouk in the arid desert region and consequently, fecundity and fertility also increased. Molecular identification underlines the predominance of *Cx. p. pipiens* form molestus and hybrids which may increase the WNV transmission [26]. Fonseca et al. [21] showed using molecular markers, that in North America 40 % of *Cx. pipiens* females have genetical characteristics of hybrids between the two European biotypes, molestus and typical pipiens (*s.s*.): they feed readily on both birds and humans thereby serving as efficient bridge vectors of WNV.

The growth and development of mosquitoes are largely determined by the environmental conditions experienced during the immature stages [27]. The quality and quantity of the larval diet shapes adult phenotypes, some of which may be important determinants of vectorial capacity [28]. The chemical and physical analysis of 10 larval breeding sites showed that *Cx. p. pipiens* larvae develop at high densities in water moderately charged in organic matter. A highly polluted breeding site can inhibit larval development. The adaptive potential of *Cx. pipiens *(*s.l.*) is remarkable; larvae are able to breed in a great variety of habitats and adults to survive in different biotopes. The two forms, pipiens and molestus, presented specific biological requirements, which led them to colonize distinct biotopes. The anautogenous pipiens form needs to blood feed on accessible hosts imposing them to breed in aboveground sites (e.g. open ditches, rain barrels,  etc.) whereas the autogenous molestus form colonizes underground sites (e.g. subways, covered wells, etc.) [29, 30]. High organic contents is mainly associated with autogenous mosquitoes [31, 32].

The molestus form can be distinguished from the pipiens form by consistent differences in the expression of facultative autogeny [32] and stenogamy [33–36]. Thus, underground sites were expected to host a higher proportion of autogenous and stenogamous females. We showed that a high proportion of anautogenous and eurygamous mosquitoes can be found in some underground sites, presumably related to the occurrence of a high proportion of hybrids or a mixed population between molestus and pipiens resulting from overflow of underground to aboveground breeding sites. Moreover, we showed that pipiens and molestus forms were mainly anautogenous/eurygamous (~100 %) and autogenous/stenogamous (78.6 %), respectively. However, we underlined that hybrids can express several phenotypes. In the same line, we observed that only six (18.7 %) of all hybrids issued from Tinerkouk have expressed an autogeneous behaviour while 81.3 % were anautogenous from the other sites. Spielman [32] demonstrated that in North America, hybrids resulting from the hybridization between autogenous and anautogenous mosquitoes can produce fertile eggs autogenously, sterile eggs and eggs with an anautogenous phenotype. We also found high densities of autogenous mosquitoes corresponding to molestus form colonizing preferentially aboveground sites; this result contrasts with observations in the Palaearctic region [37]. We also found that the proportion of hybrids (36.8 %) is higher than in Kothera et al.’s paper [38] where they found that the rate of hybrids from United States populations of *Cx. p. pipiens* ranged from 4 to 15 %. One explanation would be that in the studied areas, above and underground breeding sites are too close to each other, which may help frequent contacts and then mating between the two forms. Intriguingly, a lot of molestus form was detected in aboveground sites, which is in agreement with the observations of Nelms et al. [26].

We found a positive correlation between blood meal and egg production. Anautogenous mosquitoes are expected to be more fecund and to produce more than 400 eggs after feeding on a chicken [39, 40]. We showed that the blood meal increases the number of eggs produced (~120 eggs/mosquito) compared to mosquitoes fed on sugar only (~62 eggs/mosquito), as expected [29]. Blood feeding conditions egg production [41], adult longevity [42], oviposition [43], egg size [44] and egg-clutch size [45]. Nevertheless, blood meal does not influence fertility*.*

We found that the geographical distribution of the two forms pipiens and molestus overlaps in the same habitats in Algeria. Sympatric distribution of both forms has been already described in Europe [46–49], USA [21], Morocco [50] and Tunisia [51]. Hybrids may play the role of bridge vectors for pathogen transmission between animals and humans as this has been demonstrated in the USA for WNV [21, 52]. Hybrids were also detected in southern [47] and northern Europe [53]. Both pipiens and molestus forms are efficient vectors of West Nile and other arboviruses [54–56]. Moreover, in a previous study we showed that *Cx. p. pipiens* populations collected in Algeria were highly susceptible to infection and readily able to transmit WNV and to a lesser extent, RVFV [5].

In an anticipated result, the expression of some biological characteristics such as autogeny and stenogamy differs from northern breeding sites to southern ones, from the humid bioclimatic region in the north to the arid desert region in the south. The underground site in Tinerkouk was exposed to relatively high concentrations of dissolved organic matter and also to high temperatures compared to a similar site in El-Kala (see Table 1). Tinerkouk site also hosted *Cx. p. pipiens* expressing the highest levels of autogeny and stenogamy attributed mostly to hybrids and to a lesser extent, to molestus form as it was expected (see Table 2). It is well documented that nutritional factors [57] as well as exposure to high temperatures [58] can cause epigenetic alterations resulting in an increase of global DNA methylation. Changes in DNA methylation whose level varied inversely with gene transcription seemed to play a role in facilitating plasticity in response to environmental stress leading to micro-evolutionary changes in populations [59]. A clear example of how environment plays an important role in shaping the epigenome is represented by monozygotic twins, who are epigenetically indistinguishable early in life but with age exhibit substantial differences in epigenetic markers [60]. The effect of environment on epigenome changes is obvious even in flowering plants where vernalization requires methylation of specific histone arginine and lysine residues [61, 62], revealing a link between temperature and chromatin state. It would be tempting to speculate that autogeny and stenogamy are induced phenotypes that can be transmitted to the progeny with consequences on their vectorial potential.

## Conclusions

This study represents the first molecular evidence of *Cx. pipiens* complex forms (pipiens, molestus and hybrids) in Algeria. Our results show the simultaneous occurrence of the three biotypes, often in sympatry, both in above- and underground environments. Our findings also indicate that molestus and hybrid forms include populations that have an unusual mixture of phenotypes and differ from northern breeding sites to southern ones, indicating a possible implication of epigenetic phenomenon. These particular biological traits seem to be an adaptive process to the tough conditions in the Saharan area. Our data demonstrate a great plasticity of this complex of mosquitoes to a wide range of ecological conditions and provide valuable information for implementation of controlling methods. 

## Additional files

Additional file 1: Table S1. Major plant groups found around the breeding sites of *Culex pipiens* (*s.l.*) (DOCX 15 kb) Additional file 2: Figure S2. PCR amplification of the flanking region of the CQ11 microsatellite of *Culex pipiens* collected in an aboveground rural site in M’Sila (Algeria). One leg was used as DNA source directly in the mix. PCR products were run on a 2% agarose gel and individuals were scored according to the band size. (DOCX 146 kb) Additional file 3: Figure S1. Relation between BOC5 (Biological oxygen consumption) and mean density of *Culex pipiens* (*s.l.*) larvae. (DOCX 22 kb)
