# Supplementary material for: Ecological differentiation of members of the Culex pipiens complex, potential vectors of West Nile virus and Rift Valley fever virus in Algeria
Source: Parasit Vectors. 2016 Aug 17;9:455. doi: 10.1186/s13071-016-1725-9 (PMC4989528; doi:10.1186/s13071-016-1725-9)
Supplement: Additional file 1: Table S1. — Major plant groups found around the breeding sites of Culex pipiens (s.l.) (DOCX 15 kb) [file 13071_2016_1725_MOESM1_ESM.docx]

**Additional file 1. Table S1**. Major plant groups found around the breeding sites of *Culex pipiens s.l.*

| Site | Habitat | Breeding sites | Plant species |
| --- | --- | --- | --- |
| El-Kala | Urban | Aboveground | *Datura stramonium (*[*Solanaceae*](http://fr.wikipedia.org/wiki/Solanaceae)*); Arundo donax (Poaceae); Inula viscosa (Asteraceae); Cynodon dactylon (Poaceae); Senecio vulgaris (*[*Asteraceae*](http://fr.wikipedia.org/wiki/Asteraceae)*); Ricinus communis (Euphorbiaceae); Acanthus mollis (Acanthaceae); Euphorbia helioscopia (*[*Euphorbiaceae*](http://fr.wikipedia.org/wiki/Euphorbiaceae)*)* |
|  | Sub-urban | Aboveground | *Citrus aurantium (Bigaradeae); Picris echiodes (Asteraceae) ; Galactites tomentosa (Asteraceae); Cynodon dactylon (Poaceae); Inula viscosa (*[*Asteraceae*](http://fr.wikipedia.org/wiki/Asteraceae)*)* |
|  | Rural | Aboveground | *Mentha pulegium (*[*Lamiaceae*](http://fr.wikipedia.org/wiki/Lamiaceae)*); Galactites tomentosa (*[*Asteraceae*](http://fr.wikipedia.org/wiki/Asteraceae)*); Geranium rubertianium (*[*Geraniaceae*](http://fr.wikipedia.org/wiki/Geraniaceae)*); Bellis sylvestris (*[*Asteraceae*](http://fr.wikipedia.org/wiki/Asteraceae)*); Trifolium arvense (*[*Fabaceae*](http://fr.wikipedia.org/wiki/Fabaceae)*); Rubus ulmifolius (*[*Rosaceae*](http://fr.wikipedia.org/wiki/Rosaceae)*); Phalaris bulbosa (Poaceae); Lolium perenne (Poaceae); Senecio vulgaris (*[*Asteraceae*](http://fr.wikipedia.org/wiki/Asteraceae)*); Chrysanthemum coronarium (*[*Asteraceae*](http://fr.wikipedia.org/wiki/Asteraceae)*)* |
|  |  |  |  |
| M’Sila | Sub-urban | Aboveground | *Phragmites australis (*[*Poaceae*](http://fr.wikipedia.org/wiki/Poaceae)*); Lygeum spartum (*[*Poaceae*](http://fr.wikipedia.org/wiki/Poaceae)*)* |
|  | Rural | Aboveground | *Juniperus phoenicea (*[*Cupressaceae*](http://fr.wikipedia.org/wiki/Cupressaceae)*); Artemisia herba alba (*[*Asteraceae*](http://fr.wikipedia.org/wiki/Asteraceae)*); Arthrophytum scoparium (*[*Amaranthaceae*](http://fr.wikipedia.org/wiki/Amaranthaceae)*); Thymelaea hirsuta (*[*Thymelaeaceae*](http://fr.wikipedia.org/wiki/Thymelaeaceae)*); Lygeum spartum (*[*Poaceae*](http://fr.wikipedia.org/wiki/Poaceae)*); Salsola vermiculata (*[*Chenopodiaceae*](http://www.plantes-botanique.org/famille_chenopodiaceae)*)* |
|  |  |  |  |
| Tinerkouk | Urban | Underground | *Ammophila arenaria (*[*Poaceae*](http://fr.wikipedia.org/wiki/Poaceae)*); Phoenix dactylifera (Arecaceae)* |
