# Supplementary material for: Ecological differentiation of members of the Culex pipiens complex, potential vectors of West Nile virus and Rift Valley fever virus in Algeria
Source: Parasit Vectors. 2016 Aug 17;9:455. doi: 10.1186/s13071-016-1725-9 (PMC4989528; doi:10.1186/s13071-016-1725-9)
Supplement: Additional file 2: Figure S2. — PCR amplification of the flanking region of the CQ11 microsatellite of Culex pipiens collected in an aboveground rural site in M’Sila (Algeria). One leg was used as DNA source directly in the mix. PCR products were run on a 2% agarose gel and individuals were scored according to the band size. (DOCX 146 kb) [file 13071_2016_1725_MOESM2_ESM.docx]

**Additional file 2. Figure S2.** PCR amplification of the flanking region of the CQ11 microsatellite of *Culex pipiens* collected in an aboveground rural site in M’Sila (Algeria). One leg where used as DNA source directly in the mix. PCR products were run on a 2% agarose gel and individuals were scored according to the band size.


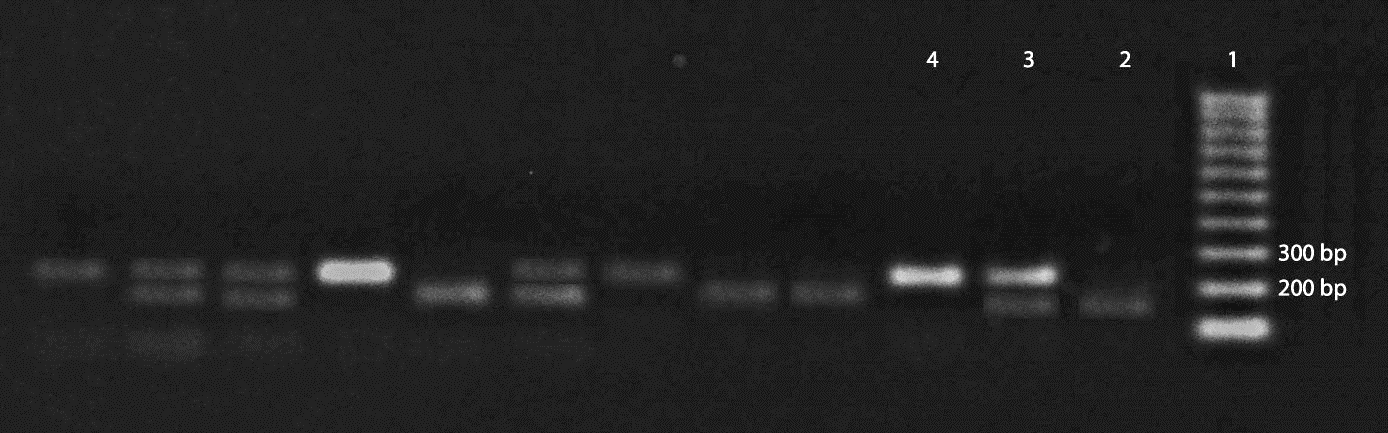


Lane 1: 100 bp size marker; Lane 2: pipiens form; Lane 3: hybrid form; Lane 4: molestus form.
