# Supplementary material for: Ecological differentiation of members of the Culex pipiens complex, potential vectors of West Nile virus and Rift Valley fever virus in Algeria
Source: Parasit Vectors. 2016 Aug 17;9:455. doi: 10.1186/s13071-016-1725-9 (PMC4989528; doi:10.1186/s13071-016-1725-9)
Supplement: Additional file 3: Figure S1. — Relation between BOC5 (Biological oxygen consumption) and mean density of Culex pipiens (s.l.) larvae. (DOCX 22 kb) [file 13071_2016_1725_MOESM3_ESM.docx]

**Additional file 3. Figure S1.** Relation between BOC5 (Biological oxygen consumption) and mean density of *Culex pipiens* *s.l.* larvae.
